# Current advancements in cellular immunotherapy for autoimmune disease

**DOI:** 10.1007/s00281-024-01034-5

**Published:** 2025-01-16

**Authors:** Corbett T. Berry, Caitlin S. Frazee, Patrick J. Herman, Sisi Chen, Anna Chen, Yvonne Kuo, Christoph T. Ellebrecht

**Affiliations:** 1 Department of Dermatology, Perelman School of Medicine, University of Pennsylvania, Philadelphia, PA USA; 2 Center for Cellular Immunotherapies, Perelman School of Medicine, University of Pennsylvania, Philadelphia, PA USA

**Keywords:** Autoimmunity, Cellular immunotherapy, Chimeric antigen receptor T cells

## Abstract

The management of autoimmune diseases is currently limited by therapies that largely suppress the immune system, often resulting in partial and temporary remissions. Cellular immunotherapies offer a targeted approach by redirecting immune cells to correct the underlying autoimmunity. This review explores the latest advances in cellular immunotherapies for autoimmune diseases, focusing on various strategies, such as the use of chimeric antigen receptor (CAR) T cells, chimeric auto-antibody receptor (CAAR) T cells, regulatory T cells (Tregs), and tolerogenic dendritic cells (TolDCs). We review recent preclinical studies and results from clinical trials that demonstrate the potential for these therapies to either deplete autoreactive cells or promote immune tolerance through broad or selective targeting of immune cell populations. Key challenges such as ensuring specificity, preventing off-target effects, and improving the longevity of therapeutic effects are discussed. The evolving landscape of cellular immunotherapies holds promise for more durable treatment responses and increased specificity for autoimmune disease treatment.

## Introduction: Autoimmunity and the rationale for cellular immunotherapies

The differentiation between self and non-self-antigens is a fundamental function of the immune system, guiding the defense against pathogens while preserving the body’s own cells. However, this delicate equilibrium can be disrupted, leading to the development of autoimmune diseases, in which the immune system erroneously targets the body’s own tissues. These disorders constitute a heterogeneous group, impacting approximately 3–10% of populations in the USA and Europe, with diversity in clinical manifestation, treatment response, and age of onset [1].

The mechanisms driving the pathogenesis of autoimmunity are multifaceted, involving B lymphocytes with their auto-antibody production and T lymphocytes with self-reactivity, underpinning the development of both organ-specific and systemic autoimmune diseases [2]. Proposed pathogenic processes are believed to arise from genetic and environmental factors, some of which include epitope spreading, bystander activation, persistent viral infections, and molecular mimicry [2, 3]. Historically, the therapeutic approach to autoimmunity has largely been dependent on a spectrum of immunosuppressive therapies, from glucocorticoids to small molecule inhibitors and advanced biological agents. Although these treatments can effectively manage symptoms and exert modulatory effects on the immune response, they are not curative. Continuous treatment is often necessary, and patients face the potential risks of serious infections and malignancy due to the long-term immunosuppression required to control their disease. The advent of biological agents has marked a significant advance, focusing on more targeted approaches to immunomodulation. Monoclonal antibodies and small molecule inhibitors offer precision in their therapeutic targets yet often fall short in disease eradication, and long-term usage may lead to a host of adverse effects. With the surge in immunotherapeutic strategies in oncology [4], the concept of harnessing the immune system has transitioned into the realm of autoimmune diseases.

In recent years, cellular immunotherapy has emerged as a groundbreaking approach, offering more than the targeted destruction of aberrant cells — as exemplified by chimeric antigen receptor (CAR) T cell therapy [5] — but also encompassing a variety of strategies designed to recalibrate the immune system’s erroneous targeting of self-tissues. This review will additionally delve into existing cellular immunotherapies, such as regulatory T cell therapy, which fosters immune tolerance, and other novel modalities like engineered T cell receptor (TCR) therapies. Each of these avenues holds the promise of redefining autoimmune disease management by selectively suppressing or modulating the maladaptive immune response while striving to retain, or even enhance, the immune system’s protective capabilities.

## Types of cellular immunotherapy for autoimmune disease

**Fig. 1:**
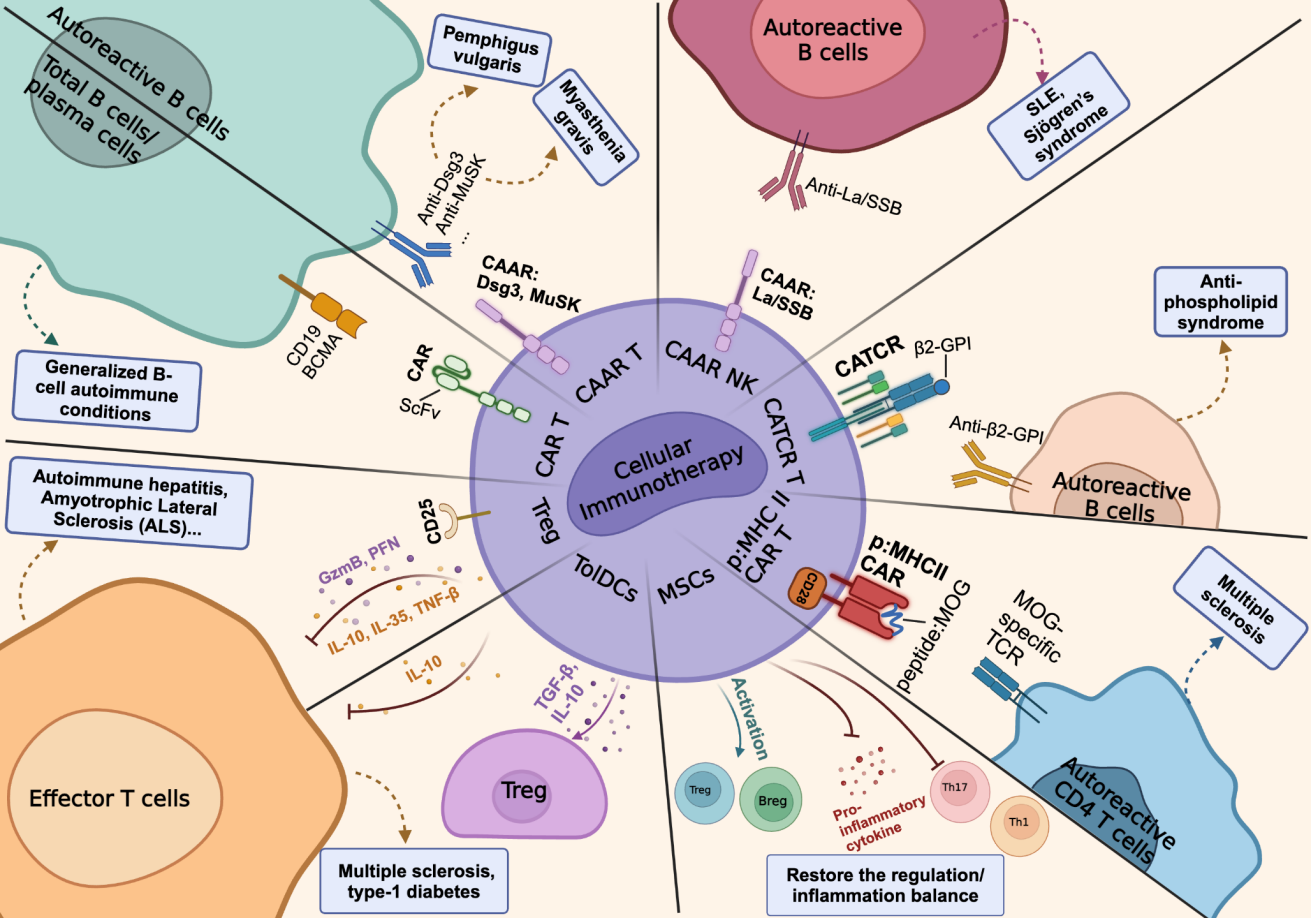
Cellular immunotherapy for the treatment of autoimmune disease. Chimeric Antigen Receptor (CAR) T cells express a CAR with single chain variable fragment (scFv) targeting CD19 or BCMA on healthy and autoreactive B cells. Anti-CD19 and BCMA are currently under investigation for B cell mediated autoimmune conditions including systemic lupus erythematosus (SLE), pemphigus vulgaris, dermatomyositis, systemic sclerosis, myasethenia gravis, and multiple sclerosis. Chimeric autoantibody receptor (CAAR) T cells express a modified CAR in which the scFv is replaced by the extracellular domain of an autoantigen. Anti-Desmoglein 3 (Dsg3) and anti-Muscle-Specific tyrosine Kinase (MuSK) are currently under investigation for pemphigus vulgaris and myasthenia gravis, respectively. Natural Killer (NK) cells have also been engineered to express CARs and CAARs to target B cell-mediated disease. Chimeric autoantigen T cell receptor (CATCR) T cells incorporate an autoantigen into a T cell receptor CD3 complex to target autoreactive B cells. CATCR targeting B cells expressing autoantibodies to beta-2-glycoprotein are currently under preclinical investigation. Peptide: Major Histocompatibility complex 2 (p: MHCII) CAR T cells specifically target autoreactive CD4 T cells by incorporating the beta chain of an MHCII ab dimer. Addition of a peptide of interest such as myelin oligodendrocyte glycoprotein (MOG) allows for selective killing of CD4 T cells recognizing auto-antigen MOG. Mesenchymal stem cells (MSCs) are multipotent cells that display immunosuppressive properties and are currently being investigated for a wide array of autoimmune conditions. Tolerogenic dendritic cells (TolDCs) are engineered DCs that exhibit immunomodulatory potential through secretion of anti-inflammatory cytokines including TGF-b and IL-10. Regulatory T cells (Tregs) generated through ex vivo expansion also have the capacity to promote tolerance through expression of immunomodulatory cytokines

In the rapidly evolving field of cellular immunotherapy, a multitude of strategies are being explored and are at various stages of preclinical and clinical development for the treatment of autoimmune disease (Fig. 1). Each approach has a common goal of leveraging a patient’s own cells for the treatment of autoimmunity. These approaches involve either enriching naturally occurring beneficial cell populations or engineering cells to specifically recognize and target pathological cells through direct cytotoxic or immunomodulatory mechanisms. Such therapies are designed to offer a more precise and potentially enduring solution to autoimmunity by directly influencing the disease course at the cellular level.

### Chimeric antigen receptor (CAR) T cells

Among the innovative avenues into cellular immunotherapy, Chimeric Antigen Receptor (CAR) T cell therapy stands out for its targeted approach and demonstrated clinical efficacy. Initially developed to treat individuals with human immunodeficiency virus [6], CAR T cell technology was later adapted for cancer treatment. Following the initial success in treating a patient with chronic lymphocytic leukemia [7], CAR T cell therapy has achieved considerable success in treating various blood malignancies, including a range of B cell leukemias and lymphomas [5]. The FDA has approved CAR T cell products that specifically target CD19 and BCMA, marking significant milestones in cancer treatment. Mechanistically, these engineered T-cells undergo ex vivo modification with a chimeric antigen receptor that contains a single chain variable fragment (scFv) targeting an antigen of interest, thus orchestrating the selective destruction of pathogenic cells (Fig. 1). By harnessing the patient’s immune system, CAR T cells offer a tailored approach that has the dual capacity to alleviate autoimmune pathology while minimizing collateral damage to healthy tissues.

Recent explorations into CAR T cell therapy’s applications in autoimmune diseases have shown promising shifts from the traditional, ongoing drug regimens to potential single-administration cures by targeting CD19 on B cells. By eliminating autoreactive B-cell clones along with all other B cells, a patient’s immune system can reset, potentially restoring self-tolerance without the interference of pathogenic clones that initiate disease [8]. Clinical trials are now actively investigating the efficacy of B cell targeting CD19 and B cell maturation antigen (BCMA) CAR T cells across a spectrum of autoimmune diseases, including systemic lupus erythematosus, myositis, pemphigus, multiple sclerosis, myasthenia gravis, and systemic sclerosis, with preliminary results indicating significant therapeutic gains (Table 1).

**Table 1:** Clinical trials utilizing cellular immunotherapies for treatment of autoimmune disease

CAR T therapy		
Target	Conditions	NCT number
CD19	Systemic lupus erythematosus (SLE)	NCT06316791, NCT05474885, NCT06310811, NCT06056921, NCT05765006, NCT06106906, NCT06106893, NCT06333483, NCT03030976, NCT06010472, NCT06316076, NCT05030779, NCT06222853, NCT06328777, NCT05846347, NCT05858684, NCT06294236, NCT05930314, NCT06189157, NCT06297408, NCT06340490, NCT05988216, NCT05869955, NCT06153095, NCT05798117, NCT06121297
	Lupus nephritis (LN)	NCT05085418, NCT06342960, NCT05938725, NCT06279923
	Multiple sclerosis (MS)	NCT06138132, NCT06279923, NCT06220201, NCT05869955
	Myasthenia Gravis (MG)	NCT06193889, NCT05828225
	Neuromyelitis optica (NMO)	NCT03605238, NCT06279923, NCT05828212
	Refractory Scleroderma	NCT05085444
	Sjogren’s Syndrome (SS)	NCT05085431
	Thrombocytopenia Alloimmune	NCT06337474
	Dermatomyositis	NCT06279923
	Refractory POEMS Syndrome	NCT05263817
	Amyloidosis	NCT05263817
	Autoimmune Hemolytic Anemia (AIHA)	NCT05263817, NCT06231368, NCT06212154
	Vasculitis	NCT05263817
	Myositis	NCT06154252, NCT05869955
	B cell-implicated autoimmune diseases	NCT05459870, NCT06152172, NCT05859997, NCT06347718
BCMA	Systemic lupus erythematosus (SLE)	NCT05846347, NCT05858684, NCT05474885, NCT06038474, NCT05030779
	Lupus nephritis (LN)	NCT05085418, NCT06277427
	Vasculitis	NCT06277427, NCT05263817
	Refractory Scleroderma	NCT05085444
	Sjogren’s Syndrome (SS)	NCT05085431
	Refractory POEMS Syndrome	NCT05263817
	Amyloidosis	NCT05263817
	Autoimmune Hemolytic Anemia (AIHA)	NCT05263817
	B cell-implicated autoimmune diseases	NCT05459870, NCT06249438
	Autoimmune diseases of the nervous system	NCT04561557
	Myasthenia Gravis (MG)	NCT04146051
CD20	Systemic lupus erythematosus (SLE)	NCT06153095
	B cell-implicated autoimmune diseases	NCT06249438
CD7	B cell-implicated autoimmune diseases	NCT05239702
BAFF-ligand	Systemic lupus erythematosus (SLE)	NCT06340750
**CAAR T therapy**		
**Target**	**Conditions**	**NCT number**
Anti-Dsg3	Pemphigus vulgaris (PV)	NCT04422912
Anti-MuSK	MuSK Myasthenia Gravis (MuSK-MG)	NCT05451212
**Treg therapy**		
NA	Type 1 diabetes mellitus (T1D)	NCT02932826, NCT03011021, NCT01210664, NCT02772679, NCT04820270, NCT05566977
NA	Rheumatoid arthritis (RA)	NCT06201416
NA	Ulcerative colitis	NCT04691232
NA	Pemphigus	NCT03239470
NA	Autoimmune hepatitis (AIH)	NCT02704338
NA	Immune Dysregulation Polyendocrinopathy Enteropathy X-linked Syndrome (IPEX)	NCT05241444
NA	Lupus nephritis (LN)	NCT02428309, NCT05566977
NA	Systemic lupus erythematosus (SLE)	NCT02428309, NCT05566977
**TolDC therapy**		
**Target**	**Conditions**	**NCT number**
NA	Multiple sclerosis (MS)	NCT05451069, NCT02618902, NCT02903537, NCT02283671, NCT04530318
NA	Neuromyelitis optica (NMO)	NCT02283671
NA	Rheumatoid arthritis (RA)	NCT03337165, NCT05251870, NCT01352858
NA	Type 1 diabetes mellitus (T1D)	NCT05207995, NCT04590872
**MSC Therapies**		
**Target**	**Conditions**	**NCT number**
NA	Autoimmune hepatitis (AIH)	NCT01661842
NA	Autoimmune pulmonary alveolar proteinosis (aPAP)	NCT06111846
NA	Chronic inflammatory demyelinating polyneuropathy (CIDP)	NCT04825626
NA	Chronic urticaria	NCT02824393
NA	Cicatrial conjunctivitis	NCT05520086
NA	Crohn’s disease	NCT01540292
NA	Diabetic nephropathy	NCT03840343, NCT04869761
NA	IgA nephropathy	NCT04342325
NA	Lupus nephritis (LN)	NCT03580291, NCT03673748, NCT04318600, NCT01539902, NCT05631717, NCT06058078
NA	Multiple sclerosis (MS)	NCT01364246, NCT00781872, NCT04749667, NCT02418325, NCT01228266, NCT02495766, NCT01895439, NCT03822858, NCT01854957, NCT02326935, NCT04823000, NCT05532943, NCT02035514, NCT04956744, NCT05116540, NCT02587715, NCT03778333, NCT01745783, NCT00395200, NCT05003388, NCT01056471, NCT02239393, NCT03326505, NCT02403947, NCT01730547
NA	Neuromyelitis optica (NMO)	NCT01364246, NCT02249676
NA	Refractory immune thrombocytopenia (ITP)	NCT04014166
NA	Rheumatoid arthritis (RA)	NCT03186417, NCT01547091, NCT03828344, NCT01663116, NCT05003934, NCT03618784, NCT03333681, NCT03691909, NCT03798028
NA	Sjögren’s Syndrome (SS)	NCT04615455
NA	Systemic lupus erythematosus (SLE)	NCT03219801, NCT03171194, NCT03562065, NCT02633163, NCT04184258, NCT04835883
NA	Type 1 diabetes mellitus (T1D)	NCT04061746, NCT01143168, NCT03912480, NCT03973827, NCT00690066, NCT02138331, NCT01686139, NCT01496339, NCT05308836, NCT03484741, NCT01157403, NCT01068951, NCT01374854

### Chimeric autoantibody receptor (CAAR) T cell

Several autoimmune diseases are directly caused by autoantibodies binding to self-antigens. These diseases are distinct because their autoantibodies directly cause disease, unlike in lupus, where for example double-stranded DNA antibodies are merely associated with the disease and do not cause symptoms when passively transferred. This understanding led to the development of chimeric autoantibody receptor (CAAR) T cell therapy, which modifies the CAR-T approach to specifically recognize and kill pathogenic autoreactive B cells while sparing the normal B cells essential for humoral immunity [9]. Currently existing CAARs utilize the transmembrane and intracellular domains of clinically-validated CARs but replaces the scFv domain with the extracellular domains of an autoantigen to target cognate autoantibody-expressing B cells [9] (Fig. 1). The first described CAAR uses a portion of desmoglein 3 (Dsg3) as its extracellular domain to treat pemphigus vulgaris (PV), an autoimmune blistering disease caused by autoantibodies against the keratinocyte adhesion protein Dsg3. In an active immune PV mouse model, Dsg3 CAAR T cells selectively eliminated anti-Dsg3 B cells, reducing anti-Dsg3 serum autoantibody titers and improving blistering [10]. Dsg3 CAAR T showed comparable efficacy to anti-CD19 CAR T in eliminating anti-Dsg3 B cells, even in the presence of soluble anti-Dsg3 antibodies [9]. A muscle-specific tyrosine kinase (MuSK) CAAR T was subsequently devised for MuSK myasthenia gravis (MG), a neuromuscular autoimmune disease caused by anti-MuSK autoantibodies [11]. Similarly, MuSK CAAR T, engineered with the full MuSK ectodomain, specifically eliminated anti-MuSK B cells in vivo and in an active immune MG mouse model. Clinical trials are now actively investigating CAAR T cell therapy in mucosal-dominant pemphigus vulgaris [12] and MuSK myasthenia gravis [13].

### CAARnatural killer (NK) cells

CAR natural killer (CAR NK) and CAAR NK cells are emerging as potential alternatives to CAR T cell therapy in the field of adoptive cell therapy due to several advantages. Engineered NK cells can be produced from various sources, such as NK92 cell lines, umbilical cord blood, and induced pluripotent stem cells (iPSCs), allowing for the creation of “off-the-shelf” products [14]. This bypasses the challenges of harvesting sufficient autologous T cells from immunosuppressed patients, a process that can delay treatment and is sometimes unfeasible. Moreover, CAR NK cells exhibit a different cytokine release profile upon activation, potentially reducing the risks of cytokine release syndrome (CRS) and neurotoxicity, severe side effects often associated with CAR T cell therapy in lymphoma and leukemia patients [15]. These attributes make engineered NK cells a promising candidate for cellular immunotherapy of autoimmune conditions, potentially leading to more accessible and less toxic treatments.

The application of this technology in autoimmunity is still in early stages but is being explored through innovative treatments such as CAAR NK cells that recognize the La/SSB protein, relevant to diseases like SLE, neonatal lupus, and Sjögren’s syndrome [16]. These specifically engineered NK cells have exhibited promising in vitro specificity and cytotoxicity against target cells. Additionally, CAAR NK and CAAR T cells directed against MN-associated antigens PLA2R1 and THSD7A have shown potential in preliminary studies, effectively targeting the implicated autoantibody-producing cells [17]. Although in the early stages of research, these findings offer a glimpse into the potential of CAAR NK cells to provide more focused and safer treatments in autoimmunity.

### Chimeric autoantigen T cell receptor (CATCR)-T cell therapy

A related concept to the CAAR T, chimeric autoantigen T cell receptor (CATCR)-T cell therapy also selectively depletes autoreactive B cells. The CATCR construct incorporates an autoantigen into a T cell receptor (TCR)-CD3 complex. The CATCR is then expressed in patient T cells, whose endogenous TCR is disrupted in parallel (Fig. 1). Mog et al. developed a β2-GPI CATCR for anti-phospholipid syndrome (APS), an autoimmune blood clotting disorder characterized by autoantibodies against beta-2-glycoprotein I (β2-GPI). Initial studies demonstrated that β2-GPI-CATCR-T-cells selectively eliminated anti-β2-GPI B cells in a dose-dependent manner in vivo [18].

### Regulatory T cells (Tregs)

Regulatory T cells (Tregs) have garnered significant attention for their potential as a novel cellular immunotherapy due to their inherent ability to maintain immunological homeostasis. Tregs achieve this by directly regulating cell behavior through either secreted cytokines, such as IL-10, IL-35, and TGF-beta, through ligation of co-inhibitor receptors on Tregs (e.g. CTLA4, PD1, TIGIT, LAG3, TIM), or through direct delivery of cytolytic enzymes like granzyme and perforin [19]. Tregs also indirectly regulate cell behavior by altering their microenvironment through either depleting extracellular ATP, expressing CD39/73 [20], or competition for the pro-inflammatory cytokine IL-2 through overexpression of CD25, the high-affinity receptor for IL-2 [19]. The concept of leveraging the regulatory functions of Tregs for developing autoimmune disease treatments represents a promising avenue of immunotherapy research, with autologous injection of Tregs isolated from patients’ blood being a popular area of investigation.

In this context, Tregs are enriched from peripheral or umbilical cord blood and then expanded ex-vivo for about 2–5 weeks to yield greater numbers of phenotypically suppressive T-cells before infusion into patients [21]. Over 150 clinical trials have used Treg infusions for autoimmune conditions, hematologic cancers, or solid organ transplants [22]. Additionally, clinical trials have been initiated for the following indications: Type 1 diabetes mellitus, autoimmune hepatitis, lupus erythematosus, pemphigus vulgaris, Crohn’s disease, ulcerative colitis, amyotrophic lateral sclerosis (ALS), Alzheimer’s disease, and COVID-19 induced acute respiratory distress syndrome [23]. These trials have yielded variable success: Thonhoff et al. [24] found a slower progression rate of both early and late-stage ALS assessed via the Appel ALS score, while Dall’era et al. found no clinical improvement in SLE patients [25].

There have also been advancements in genetically engineering CAR Tregs ex-vivo to localize the suppressive cells to the site of autoimmune manifestation. Two popular strategies have emerged to direct Treg cells to their therapeutic site. The first is to use a CAR signaling domain containing an scFv that directs the Tregs to an antigen present on the affected tissue [26]. Ellis et al. engineered non-human primate alloantigen Bw6 + CAR Tregs and demonstrated their ability to maintain suppressor function and traffic to the site of transplanted allogeneic beta islet cells [27]. The other technical approach is to use an engineered TCR that identifies a peptide: MHC specific to affected cells [28]. These targeted approaches for engineering CAR Tregs offer an opportunity to promote selective tolerance and reduce potential adverse inflammatory events due to Treg conversion to pro-inflammatory Th17 T cells [29]. CAR Tregs are currently being investigated on a preclinical level to treat rheumatoid arthritis, type-1 diabetes, asthma, multiple sclerosis, inflammatory bowel disease, vitiligo, and hemophilia (Table 1). There are also active clinical trials investigating the use of CAR Tregs for kidney transplants and graft-versus-host disease.

### Peptide: Major histocompatibility complex 2 (p: MHCII) CAR T

Recent developments have introduced a new level of precision in targeting CD4 + T cells, critical mediators of autoimmune disease [30]. Peptide-MHC class II (p: MHCII) CAR therapies are designed to precisely target and eliminate autoreactive CD4 + T cells, key players in the pathogenesis of many autoimmune conditions. The p: MHCII CAR is composed of a peptide linked to the β chain of an MHCII αβ dimer, which is attached to an intracellular signaling domain used in clinically-validated CARs [31]. Yi et al. have engineered a MOG p: MHCII CAR for multiple sclerosis (MS), targeting the myelin-specific inflammatory CD4 + T cells that are pivotal in disease progression [32]. MOG is expressed in the myelin-producing oligodendrocytes in the CNS, and MOG-specific pro-inflammatory CD4+ T cells have been identified in MS patients [31]. In a mouse model, this CAR T cell therapy prevented the onset of MS when directed at high-affinity MOG-specific T cells, reversing the disease upon targeting both high and low-affinity MOG-specific T cells.

Similarly, for rheumatoid arthritis (RA), Whittington et al. created a p: MHCII CAR with HLA-DR1, a DR allele with an increased susceptibility to RA [33]. Whittington et al. crafted a CAR T cell with HLA-DR1 loaded with a type II collagen peptide, successfully lysing collagen-specific CD4 + T cells in a mouse model, resulting in a reduction of both the autoimmune response and the incidence and severity of RA symptoms. These approaches exemplify the potential of p: MHCII CARs to mitigate autoimmune diseases by specifically addressing the underlying autoreactive T cells and could represent a paradigm shift in autoimmune therapy. However, there are challenges with the p: MHCII CAR approach, as HLA matching would be required, and reactivity against the same autoreactive peptides differs across patients.

### Tolerogenic dendritic cells

Dendritic cells (DCs) act as a crucial link connecting the innate and adaptive arms of the immune system. Beyond their well-known role in antigen presentation, DCs can be engineered to adopt a tolerogenic state through a range of strategies [34]: techniques such as ex vivo cultivation and in vivo targeting with immunomodulatory agents, including antisense oligodeoxyribonucleotides, dexamethasone, rapamycin, IL-10, and GM-CSF, enable the programming of DCs into a unique functional state [35–37]. These tolerogenic DCs (TolDCs) present a hybrid phenotype between their unactivated precursors and fully matured counterparts. This intermediate state is characterized by distinct migratory patterns, secretion of anti-inflammatory cytokines (e.g. TGF-beta, IL-10, indoleamine 2 3-dioxygenase, and Fas ligand), and induction of immune tolerance in T cells. Current clinical trials employing TolDCs are exploring treatments for autoimmune diseases such as rheumatoid arthritis, multiple sclerosis, and type-1 diabetes. Some early trials have shown promising outcomes, such as the reduction of inflammatory cytokines and a rise in the ratio of Tregs to effector T-cells in RA patients [37, 38], though not all trials have translated into clinical improvements. This suggests that fine-tuning the administration techniques of tolDCs may be important to enhancing their therapeutic impact.

### Mesenchymal stem cells

Mesenchymal stem cells (MSCs) are multipotent cells that can be used for immunomodulating and regenerative therapies [39]. They can be isolated from the umbilical cord, Wharton’s jelly, bone marrow, adipose tissue, teeth, and menstrual fluid and then expanded ex vivo for future injection into patients [40]. MSCs display immunosuppressive abilities, have low immunogenicity and interaction with T cells, can localize to damaged tissue, and interact with their local environment to promote tissue regeneration. MSCs can also differentiate into numerous cell types including osteoblasts, chondroblasts, and adipocytes in vivo. Given this range of abilities and approval for use in cancer therapies, they are a promising therapeutic for autoimmune patients. Currently, no MSC-based therapeutics have received regulatory approval for autoimmune diseases. However, clinical trial data for this approach, though heterogeneous, indicate that MSC therapies may be effective for conditions such as RA, MS, T1DM, SLE, Sjogren’s syndrome, and IBD by promoting Treg differentiation, decreasing pro-inflammatory cytokines production, and influencing T cell proliferation and function. Few adverse side effects have been reported from MSC therapies [41].

## Recent clinical progress in autoimmune disease cellular immunotherapy

### Systemic lupus erythematosus (SLE)

Pioneering studies and follow-up case series provide compelling evidence for the potential of CD19 targeting CAR T cell therapy to induce sustained remission in patients with refractory SLE, reframing the understanding and approach to treatment in autoimmune pathology. Initial findings from a single case study reported on a young adult female with severe, refractory SLE who received lymphodepleting fludarabine followed by low dose (10^6^ cells per kg) autologous CD19 CAR T cell therapy [42]. Despite a recalcitrant clinical course despite multiple lines of therapy — including high-dose glucocorticoids, cyclophosphamide, mycophenolate mofetil, tacrolimus, belimumab, and rituximab— she experienced significant clinical and serological improvements post CAR T cell treatment. Rapid expansion of CAR T cells was observed following infusion which was accompanied by complete but only transient B cell depletion. Clinically, this translated into the resolution of proteinuria, normalization of serum complement levels, and a reduction of disease activity SLEDAI scores to zero.

Subsequent case series, including five patients with SLE, provided a more nuanced view of the therapy’s impact [43]. These patients, each with active SLE affecting multiple organ systems and unresponsive to diverse immunosuppressive regimens, demonstrated similar outcomes following CD19 CAR T cell infusion. Remarkable reductions in anti-double-stranded DNA antibodies were noted, alongside normalized complement levels and marked clinical improvements. Of note, the observed decline in anti-dsDNA antibodies in some patients with high levels of these antibodies surpassed the expected rate of typical IgG catabolism (half-life of 21 days). This discrepancy may be partially explained by patients’ proteinuria in the setting of lupus nephritis but could suggest potential non-linearity in the assay used and/or hints at possible cooperative binding of IgG to dsDNA. Therefore, optimization of these assays is likely warranted, given that such pronounced reductions have not been observed with other treatment modalities. Additional efforts to identify accurate biomarkers for monitoring therapeutic efficacy are underway and remain a crucial objective for the field [44]. These studies also offered insights into the kinetics of CAR T cell expansion and B cell depletion, with only transient B cell depletion and a subsequent reconstitution phase characterized by a predominance of naïve B cells — a finding that may indicate an “immune reset”. Moreover, the duration of B cell depletion does not appear to last longer than after treatment with traditional anti-CD20 monoclonal antibodies (e.g. rituximab), suggesting that the quality (as opposed to duration) of the B cell depletion may play a more important role than previously appreciated. This may reflect more effective reduction of B cells localized to secondary lymphoid compartments or the effective depletion of an early B cell progenitor population that does not express CD20. Notably, sustained remission was observed without relapses during the follow-up period, which for some patients extended up to 29 months.

An additional follow-up study extended these observations, providing long-term data on the feasibility, safety, and efficacy of CD19 CAR T cell therapy [45]. A group of eight patients with severe SLE, unresponsive to various conventional therapies, exhibited prolonged clinical remission, with all achieving DORIS remission and successfully discontinuing all immunosuppressive drugs. B cell aplasia lasted an average of 112 days, after which a naïve B cell predominance was observed. This finding reinforces the concept that the therapy not only targets immediate pathogenic B cells but may also induce a broader immunological change, potentially fostering a more regulated and less autoreactive immune system. Importantly, the therapy’s tolerability was substantiated by a safety profile showing only mild cytokine release syndrome and no severe adverse events, challenging the often-held notion that CAR T cell therapy is invariably associated with significant toxicity.

The promising results from recent studies suggest that CD19 CAR T cell therapy may offer durable remission in SLE. With no severe adverse effects reported and persistent serological remission observed, this therapy hints at a significant shift in SLE’s disease trajectory. As such, CD19 CAR T cell therapy is poised to transition from an experimental approach to a viable treatment for SLE, potentially revolutionizing autoimmune disease management. Nonetheless, additional comprehensive clinical trials and ongoing surveillance data are vital to confirm its long-term safety and efficacy.

### Autoimmune myositis

With promising results in SLE, researchers extended the application of CD19 CAR T cells to myositis, particularly anti-synthetase syndrome, which tends to be highly refractory to traditional treatments [46]. The initial report of treatment of anti-synthetase syndrome with CD19-targeting CAR T cells involved a 41-year-old patient with persistent myositis and interstitial lung disease refractory to high-dose glucocorticoids and immunosuppressants, including rituximab and azathioprine [47]. Following CD19 CAR T cell infusion, the patient exhibited dramatic improvement in muscle strength and endurance, reductions in creatinine kinase levels and anti-Jo-1 antibodies, and complete resolution of myositis-related lesions on MRI. A second 41-year-old patient, experiencing similar resistance to treatment, received the CD19-targeted CAR T cell therapy but faced an initial increase in myalgia and creatinine kinase levels post-infusion [48]. Subsequent treatment adjustments, including the introduction of mycophenolate mofetil to target CD8 + effector memory T cells, led to a clinical improvement and sustained disease remission.

In addition to these cases, CAR T cell therapy has been more recently used for other forms of myositis. A 25-year-old patient with immune-mediated necrotizing myopathy (IMNM) was treated with BCMA CAR T cell therapy [49]. IMNM is characterized by severe muscle weakness and high levels of creatine kinase, often unresponsive to standard treatments [50]. Post-treatment with BCMA CAR T cells, the patient experienced sustained reductions in pathogenic autoantibodies and a marked improvement in clinical symptoms, including muscle strength and a reduction in serological disease markers. Furthermore, single-cell analysis post-therapy revealed a broad and lasting reprogramming of the immune system, suggesting that the therapy’s effectiveness extends beyond the direct depletion of pathogenic B cells, potentially offering a comprehensive strategy for treating autoimmune myositis. Most recently, a case series reported its use in three patients with idiopathic inflammatory myositis (IIM) [45]. All patients achieved a major clinical response, with the normalization of creatine kinase levels within three months and an improvement in muscular and extramuscular disease activity. This effect persisted throughout the follow-up, with one patient reaching 18 months post-treatment, highlighting the potential of CAR T cell therapy for sustained disease control without ongoing immunosuppression.

### Systemic sclerosis

In the evolving landscape of SSc management, CD19-targeted CAR T cell therapy has also emerged as a potentially groundbreaking treatment. Systemic sclerosis (SSc) is an autoimmune disease characterized by fibrosis of the skin and internal organs, vasculopathy, and dysregulated immune responses [51]. This chronic condition can lead to debilitating outcomes and presents significant treatment challenges when the disease progresses despite standard therapies [52]. The first report in 2023 detailed the case of a 60-year-old male with severe diffuse cutaneous SSc with cardiac and pulmonary involvement who had not responded to immunosuppressive agents like methotrexate and mycophenolate mofetil [53]. Post-CAR T cell therapy, a robust expansion of the engineered T cells and the subsequent complete B cell depletion were achieved. Although only a modest effect on skin fibrosis was observed, treatment led to a significant reduction of myocardial fibrosis and an improved EUSTAR activity index. A subsequent case of a 38-year-old woman with rapid progressive Scl70 + systemic sclerosis-associated interstitial lung disease (SSc-ILD), resistant to aggressive treatment regimens, provided further evidence for the efficacy of CD19 CART cells [54]. Infusion of third-generation CD19 CAR T cells followed by re-initiation of combination immunosuppressive therapy with mycophenolate and nintedanib led to pulmonary function improvement and regression of skin fibrosis, evidenced by PET/CT imaging showing decreased fibrosis. More recently, a larger case series included four SSc patients treated between February 2021 and May 2023, showcasing the broader applicability of CD19 CAR T cell therapy [45]. These patients, with persistent disease activity despite numerous immunosuppressive therapies, experienced rapid expansion of CAR T cells and complete B cell depletion. All patients showed a decrease in the EUSTAR activity index without severe adverse effects. These cases illustrate that CD19 CART cells may be an effective approach for improving clinical outcomes in severe refractory SSc, especially in those with cardiac and pulmonary involvement.

### Pemphigus vulgaris

Pemphigus vulgaris (PV) is an autoimmune disease characterized by painful blisters on the skin and mucous membranes [55]. Autoantibodies against the keratinocyte adhesion proteins desmoglein 1 (Dsg1) and desmoglein 3 (Dsg3) drive PV pathology. There are two main subtypes of pemphigus vulgaris: (1) mucosal PV, characterized by mucosal lesions caused by anti-Dsg3 autoantibodies, and (2) mucocutaneous PV, characterized by both mucosal and skin lesions caused by both anti-Dsg3 and anti-Dsg1 autoantibodies [55]. PV is typically treated with corticosteroids, steroid-sparing immunosuppressants (e.g. mycophenolate mofetil, azathioprine), and the B-cell depleting anti-CD20 monoclonal antibody rituximab. However, while remission is possible in 50–75% of patients [56], the majority require long-term treatment leading to increased risks of severe adverse events due to prolonged immunosuppresion [57]. This underscores the need for new therapies that effectively eliminate the autoantibody-producing cells underlying PV pathophysiology. To address these issues, a Dsg3-CAAR which selectively targets and destroys B cells specific for Dsg3 was developed and has shown promise in preclinical studies by specifically lysing anti-Dsg3 B cells, offering a step forward from broad immunosuppression to focused treatment.

Dsg3 CAAR T cells, representing the first cellular immunotherapy for pemphigus vulgaris (PV), are currently under investigation in a Phase 1 trial [12] for patients with mucosal-dominant PV. Preliminary data demonstrated that, when used without preconditioning, Dsg3 CAAR T is well-tolerated and is not associated with dose-limiting toxicities [58, 59]. Further trial data indicate that a refined preconditioning regimen may enhance the persistence of CAAR T cells, an avenue being explored to potentially bolster the therapy’s effectiveness [58]. These developments mark a critical phase in the application of cellular immunotherapy for autoantibody-mediated diseases, with Dsg3 CAAR T providing a targeted approach to address the underlying pathophysiology of PV.

### Myasthenia gravis

Myasthenia gravis (MG) is an autoimmune disorder mediated by autoantibodies that disrupt the communication between nerve and muscle at the neuromuscular junction, leading to generalized or localized weakness in the voluntary muscles [60]. The two primary pathogenic autoantibodies target the acetylcholine receptor (AChR) and muscle-specific kinase (MuSK) on the postsynaptic muscle cell membrane [61]. MuSK is involved in the formation of AChR clusters, which bind the neurotransmitter acetylcholine to initiate muscle contraction. Anti-AChR is detected in 80–85% of patients, and anti-MuSK in 1–10% [62–64]. Standard MG treatment includes cholinesterase inhibitors, corticosteroids, steroid-sparing immunosuppressants, and the B-cell-depleting anti-CD20 monoclonal antibody rituximab [61]. Despite the improvement in prognosis provided to many MG patients, 10–20% of patients do not achieve a satisfactory response or are intolerant to conventional treatment [65]. This highlights the need for new therapies that effectively eliminate the autoantibody-producing cells underlying MG pathophysiology. To address this, a MuSK CAAR T for MuSK MG was developed and engineered with the full MuSK ectodomain, showing efficacy in preclinical models similar to CD19 CAR T cells [11].

MuSK CAAR T treatment is being evaluated in a phase 1 clinical trial [13] in MuSK MG patients, aimed at establishing the safety and efficacy of therapy in the presence and absence of preconditioning therapy with cyclophosphamide and fludarabine. Concurrently, several CAR T therapies are progressing through clinical trials for generalized MG. Kyverna Therapeutics’ fully human CD19 CAR with a CD28 costimulatory domain, tested for safety and efficacy in lymphoma [66], enters a Phase 2 trial [67] with a cyclophosphamide and fludarabine lymphodepletion regimen. Meanwhile, Cabaletta Bio’s fully-human CD19 CAR with a 4-1BB costimulatory domain will soon initiate a Phase 1 and 2 trial alongside cyclophosphamide and fludarabine lymphodepletion conditioning regimen. Zhejiang University’s CD19 CAR is also a clinical exploration for refractory MG [68]. In parallel, BCMA-targeting CAR T cell therapies, such as Cartesian Therapeutics’ Descartes-08, are being explored due to their preferential action on autoantibody-producing plasma cells, which play a crucial role in MG pathophysiology. Descartes-08, an mRNA-based CAR requiring no preconditioning and allowing for controlled pharmacokinetics, has shown promising results in an early-phase trial [69] with significant reductions in disease severity scales and autoantibody levels. Descartes-08 was shown to be safe and well-tolerated with no dose-limiting toxicities, including no CRS and neurotoxicity [70]. All seven patients receiving six weekly infusions of Descartes-08 experienced clinically meaningful decreases in all four standard MG disease severity scales (MG Composite, MG Activities of Daily Living, Quantitative MG scores, and Quality of Life 15-revised) up to 9 months of follow-up. At 12 months follow-up, two out of seven participants relapsed; one patient opted for re-treatment, then experienced improvement in clinical scores maintained at 6 months of follow-up [71]. All three participants with detectable anti-AChR autoantibodies at baseline exhibited reductions that were maintained at month 12. Lastly, IASO Bio’s fully-human BCMA CAR, CT103A, previously affirmed for multiple myeloma [72], is being evaluated in a Phase 1 trial [73] for various antibody-mediated nervous system diseases, including MG. These developments underscore an era of precise cellular immunotherapies poised to transform MG management, contingent upon the affirmation of safety and efficacy in ongoing trials.

### Multiple sclerosis

MS is a chronic inflammatory disease of the central nervous system, characterized by demyelination and affecting around 2.5 million people globally [74]. It manifests with a spectrum of neurological symptoms, such as muscle weakness, coordination and balance issues, visual disturbances, and cognitive dysfunction. The disease follows a relapsing or progressive course, driven by autoreactive T cells against the myelin sheath. Tregs dysfunction, B cell mediated effects, and innate immune cell involvement have all been implicated in its pathogenesis, with recent data also suggesting a correlation with Epstein-Barr virus infections [75]. Standard treatments for MS include corticosteroids for acute flares, other immunosuppressive agents like teriflunomide and dimethyl fumarate to slow disease progression, and monoclonal antibodies such as ocrelizumab (anti-CD20) [76], rituximab (anti-CD20) [77], and natalizumab (anti-alpha 4 integrin) [78]. However, while these therapies can be effective, they are not universally successful for all patients and carry risks, including severe infections.

In response to the challenges presented by MS, specialized cellular immunotherapies are now being explored. Preclinical successes of tolDCs, loaded with MS-specific antigens, have led to at least three ongoing clinical trials examining their efficacy and safety [79]. Unmodified ex vivo expanded Tregs have been safely administered to patients with relapsing-remitting MS, yet conclusive data on their clinical efficacy is outstanding [80]. MSCs, evaluated in numerous clinical trials for chronic and relapsing MS, have shown variable results. While many trials failed to demonstrate clinical efficacy, some reported patient improvements. These trials, disparate in their protocols, culminated in a coordinated multinational phase II MESEMS trial: this concluded that MSCs were well tolerated but did not significantly reduce inflammation in active MS cases [81]. Further, specific cellular approaches, such as the development of Myelin Basic Protein (MBP) CAAR T cells, have been explored for their potential to target the autoimmune components of MS [82].

### Autoimmune coagulopathies: Hemophilia a and immune thrombocytopenic purpura

Hemophilia A is an inherited bleeding disorder that causes a deficiency in the coagulation factor VIII. The standard of care for hemophilia A patients is intravenous FVIII replacement therapy, but 20–30% of patients produce neutralizing anti-FVIII antibodies that cause treatment resistance [83]. This finding led to the development of Factor FVIII (FVIII) CAAR T as a potential prophylactic treatment for Hemophilia A patients with neutralizing anti-FVIII antibodies [84]. The FVIII CAAR comprises the A2 and C3 domains of FVIII. FVIII CAAR-expressing CD8 T cells specifically killed anti-FVIII B cell hybridomas in a dose-dependent manner in vivo and in vivo. FVIII CAAR T also prevented anti-FVIII antibody formation in a mouse model of hemophilia [84].

### NMDAR encephalitis

N-methyl-D-aspartate receptor (NMDAR) encephalitis, a severe neuropsychiatric condition marked by autoimmune attacks on neural receptors, has seen promising advances with the development of NMDAR CAAR T therapy [85]. This innovative treatment utilizes the extracellular domains of the NMDAR, GluN1, and GluN2B, to precisely target and eliminate the autoantibody-producing B cells. In preclinical studies, these CAAR T cells have demonstrated the ability to eliminate B cells bearing anti-NMDAR receptors without affecting other cells, a crucial step in preventing collateral damage. Their efficacy was further evidenced in a mouse model incorporating human B cells from patients; the treatment not only depleted the problematic B cells but also significantly reduced the harmful antibodies in both serum and brain tissue, all without any discernible off-target effects. This represents a major step in treating NMDAR encephalitis, offering a targeted approach that mitigates the disease while preserving healthy brain function.

## Overcoming challenges in cellular immunotherapies

### Improving therapeutic specificity

While progress has been achieved in unraveling the etiological factors of autoimmune diseases, particularly those attributable to specific autoantibodies, the full therapeutic potential of cellular immunotherapies remains to be seen. This progress underscores a commitment to translating insights from immunological research into clinical practice, with CD19-targeted CAR T cell therapies marking a significant and impressive milestone. Despite this advance, the efficacy of such targeted treatments is still under rigorous scrutiny, with a comprehensive understanding of selective targets for broader autoimmune spectra yet to be achieved. The development of CAAR T cells represents a methodical approach to this challenge, offering a strategy to target pathogenic B cell clones with greater specificity. This focus on precision medicine reflects the ongoing dedication to mitigate the risks associated with generalized immunosuppression and move toward more refined and safer therapeutic options. As research continues, the identification of specific antigens associated with autoimmune disorders is paramount, paving the way for the next generation of cellular immunotherapies designed to improve patient outcomes.

### Preconditioning and immunomodulation

Lymphodepleting chemotherapy has been the standard pretreatment in all FDA-approved CAR T therapies for leukemia and lymphoma, and successful treatment of autoimmune diseases with CD19-targeting CAR T cells included a lymphodepleting pre-conditioning regimen [43, 45, 47, 48]. While ongoing Dsg3 CAART trials incorporate a condition without preconditioning, future clinical trials should incorporate control groups that forgo pre-conditioning regimens. This will allow for a clearer comparison of the therapeutic effects of the pre-treatment and the efficacy of low-dose CAR T therapy. Additionally, even with preconditioning, there are still variable results from these therapies, resulting in the need for some patients to reinitiate immunosuppressants [48, 54]. Given the possibility of unsuccessful treatments with CAR T therapies, the question of whether the exorbitant costs and patient risks are justifiable must be considered.

### CAR T cell therapy-associated toxicities

CRS is a well-documented risk of CAR T cell therapy, manifesting as a systemic inflammatory response marked by a surge of cytokines from proliferating T cells [86] resulting in fever, hypotension, and potentially life-threatening complications such as multiple organ dysfunction syndrome [87–90]. Tocilizumab, targeting the IL-6 receptor, has been the mainstay treatment for severe CRS cases, mitigating the cytokine-driven cascade [91]. Thus far, clinical trials for autoimmune disease have only reported grade I CRS following CAR T cell treatment, likely due to the decreased burden of target cells compared to lymphoma and leukemia patients. No CRS has been observed to date after CAAR T cell therapy. Additionally, Immune effector Cell-Associated Neurotoxicity Syndrome (ICANS) can result from an influx of cytokines affecting the nervous system, but additional trials are needed to assess the incidence of ICANS following CAR T cell therapy for patients with autoimmune disease. To date, no cases of ICANS after CAR T cell therapy for autoimmunity has been reported.

Recent concerns about CAR T cell therapies have emerged due to reports of T cell lymphomas after treatments for hematologic malignancies [92]. These concerns have prompted a reevaluation of the risk profile of CAR T cell therapy, particularly for cancer patients pre-treated with mutagenic chemotherapies and those with hematologic B-cell neoplasms who may inherently have a higher predisposition to T cell neoplasms. Despite these concerns, a recent study reported only a single occurrence of T cell lymphoma following anti-CD19 CAR T cell treatment, indicating a relatively low incidence of such malignancies post-therapy. In a cohort of 449 patients treated at the University of Pennsylvania, only one case of Tcell lymphoma was observed [93].

Given the potential differences in clonal hematopoiesis and less intense pre-treatment regimens in autoimmune patients, these risks may not be directly applicable to individuals with autoimmune diseases. However, the possibility of T-cell malignancy necessitates a cautious approach in implementing cellular immunotherapies for autoimmune diseases, especially since autoimmune patients tend to have a more favorable prognosis compared to many patients with lymphomas and leukemias. Careful risk assessment is crucial, especially when balancing these risks against the potentially transformative benefits of CAR T cell treatments.

### Manufacturing cellular immunotherapies

Due to the current requirement of autologous cells in cellular immunotherapies, there are numerous logistical challenges surrounding scaling up their lengthy and expensive manufacturing processes [94]. However, some manufacturing technologies are being developed to bridge these gaps and shorten the ex vivo culturing process, such as T-charge, which utilizes a CAR T cell expansion process occurring primarily in vivo by engineering the CAR T cells to maintain stemness and limit exhaustion [95]. By expanding the CAR T cell population within the patient, the manufacturing process is streamlined, taking less than two days to prepare the final product [96]. T-Charge is currently being studied in first-in-human clinical trials. Additionally, a lentivector was recently designed that can specifically target and activate T cells to bypass T cell selection, potentially allowing for a same-day closed manufacturing process of autologous CAR therapies [97].

Traditionally, CAR T cells rely on viral delivery systems to insert genetic material into the host genome; however, due to the risks associated with viral vectors, there is a high cost to maintain good manufacturing practice (GMP) grade production and handle regulatory burdens. Therefore, non-viral delivery platforms are a potential avenue to circumvent these limitations, with potentially decreased manufacturing costs and immunogenicity. These non-viral products can be delivered through electroporation, liposomes, or other nanoparticle-based transfection methods [98]. For instance, lipid nanoparticles (LNPs) can be used for mRNA delivery of CAR constructs, resulting in transient CAR expression [99, 100]. These LNPs could deliver CAR RNA in vivo, bypassing the ex vivo T cell manufacturing process; however, this transient CAR expression could necessitate multiple rounds of LNP doses for treatments.

Another potential alternative to current autologous cellular therapies is “off-the-shelf” or allogeneic CAR T cells. Rather than manufacturing unique CAR T cells for each patient, these CARs would be produced with T cells from healthy donors. This strategy could address many of the challenges surrounding autologous therapies, such as high production costs and manufacturing times delaying treatments, as well as limiting the risk of product contamination by pathogenic cells [101]. However, allogeneic treatments risk rejection of donor CAR T cells by the host’s immune system, as well as causing graft-versus-host disease (GvHD), where donor cells recognize the patient’s healthy cells as foreign, attacking them. Hu et al. genetically modified CAR T cells derived from healthy donors to reduce GvHD and to avoid the host’s immune system [102]. To limit the risk of GvHD, the T cell receptor (TCR) and associated CD3 genes were disrupted; to reduce host rejection, the human leukocyte antigen (HLA) was depleted, and an NK cell inhibitory receptor (NKi) was introduced. In a phase I clinical trial, there was no dose-limiting toxicity, GvHD, ICANS, or severe CRS observed due to these allogeneic CAR T cells [103]. Further investigation is necessary, but this study established the potential for allogeneic CAR T cell therapies in the future.

## Conclusion

In conclusion, the landscape of cellular immunotherapies for autoimmune disorders is characterized by a rapid evolution of targeted strategies, as evidenced by rapidly increasing numbers of clinical trials and the preliminary successes therein. Here, we reviewed the salient advances in the field, emphasizing the potential of such therapies to instigate durable remission. Clinical data, albeit preliminary, offer an optimistic forecast for these interventions, substantiated by robust preclinical foundations. Nonetheless, the realization of these therapies as universal modalities for autoimmune conditions is tempered by intrinsic complexities, including the need for targeted antigen discovery and the potential requisite for concurrent immunosuppression. The justification of such therapies, in light of their associated risks and economic implications, remains a subject for deliberation; however, the power of these approaches, as they continue to develop, is apparent.

## Data Availability

No original datasets were created for this review.
